# Reduction of relative centrifugation force within injectable platelet-rich-fibrin (PRF) concentrates advances patients’ own inflammatory cells, platelets and growth factors: the first introduction to the low speed centrifugation concept

**DOI:** 10.1007/s00068-017-0767-9

**Published:** 2017-03-10

**Authors:** J. Choukroun, S. Ghanaati

**Affiliations:** 1 Private Practice, Pain Therapy Center, Nice, France; 2 Department for Oral, Cranio-Maxillofacial and Facial Plastic Surgery, FORM (Frankfurt Orofacial Regenerative Medicine) Laboratory, University Hospital Frankfurt Goethe University, Theodor-Stern-Kai 7, 60590 Frankfurt am Main, Germany

**Keywords:** Inflammation, Leukocytes, Platelets, Platelet-rich fibrin, Tissue engineering, Pain, Centrifugation, A-PRF+, I-PRF

## Abstract

**Purpose:**

The aim of this study was to analyze systematically the influence of the relative centrifugation force (RCF) on leukocytes, platelets and growth factor release within fluid platelet-rich fibrin matrices (PRF).

**Materials and methods:**

Systematically using peripheral blood from six healthy volunteers, the RCF was reduced four times for each of the three experimental protocols (I–III) within the spectrum (710–44 g), while maintaining a constant centrifugation time. Flow cytometry was applied to determine the platelets and leukocyte number. The growth factor concentration was quantified 1 and 24 h after clotting using ELISA.

**Results:**

Reducing RCF in accordance with protocol-II (177 g) led to a significantly higher platelets and leukocytes numbers compared to protocol-I (710 g). Protocol-III (44 g) showed a highly significant increase of leukocytes and platelets number in comparison to -I and -II. The growth factors’ concentration of VEGF and TGF-β1 was significantly higher in protocol-II compared to -I, whereas protocol-III exhibited significantly higher growth factor concentration compared to protocols-I and -II. These findings were observed among 1 and 24 h after clotting, as well as the accumulated growth factor concentration over 24 h.

**Discussion:**

Based on the results, it has been demonstrated that it is possible to enrich PRF-based fluid matrices with leukocytes, platelets and growth factors by means of a single alteration of the centrifugation settings within the clinical routine.

**Conclusions:**

We postulate that the so-called low speed centrifugation concept (LSCC) selectively enriches leukocytes, platelets and growth factors within fluid PRF-based matrices. Further studies are needed to evaluate the effect of cell and growth factor enrichment on wound healing and tissue regeneration while comparing blood concentrates gained by high and low RCF.

## Introduction

In recent years, several concepts have been introduced for clinically relevant tissue engineering by means of minimally invasive approaches. Currently, well-accepted models include pure biomaterial application [1–4] and the combination of biomaterials with autologous bone marrow aspirates [5, 6] or recombinant bone morphogenetic proteins (BMPs) [7, 8]. Although less invasive than autologous bone transplantation, the previously mentioned minimally invasive bone marrow aspirate methodologies are still reserved for only experienced surgeons, as these methods can also be associated with complications such as pain, infection, and damage to the adjacent organs during bone marrow retrieval. Furthermore, the optimal growth factor concentration needs to be determined for the use of BMPs.

To resolve these issues, concentrates from the peripheral blood have been recently proposed as potential supporters in complex tissue engineering. In this context, several blood concentrate systems were introduced such as plasma-rich growth factors (PRGF) [9] and platelet-rich plasma (PRP) [10]. Both systems require the addition of non-autologous anticoagulants to generate fluid blood concentrates after centrifugation [9]. In addition to anticoagulants, multi-step centrifugation is needed to obtain PRP [11]. Moreover, PRGF and PRP focus on the advantages of platelets and their released growth factors to support tissue regeneration in different medical fields and aim to eliminate leukocytes from the final blood concentrates [12].

The introduction of platelet-rich-fibrin (PRF) in 2001 exemplified the first step toward the generation of blood-derived PRF matrices that do not require additional anticoagulation agents [13]. The centrifugation within a specific glass-based tube leads to the activation of the physiological coagulation cascade and, thus, the formation of a fibrin clot enriched with cells within the peripheral blood i.e., platelets and leukocytes.

Recent developments in cellular and molecular biology and continuous research have increased the understanding of the wound healing and regeneration processes. Thus, the crucial role of leukocytes and their subgroups as main protagonists to modulate the various phases during wound healing became obvious [14]. In addition, platelets as well as the fibrin network are known to play a major role in the wound healing process [15]. Leukocytes participate in angiogenesis and lymphogenesis, whereas the fibrin network is a key player in the early stages of wound healing rendering its synergistic effects with platelets and its function as a reservoir of cytokines [16–18].

Nevertheless, to generate PRF the application of high relative centrifugal forces (RCF) (708 g) during the centrifugation process was described as essential. Furthermore, previous histological analyses of the PRF clot demonstrated that platelets and inflammatory cells within the fibrin scaffold accumulate mainly in the proximal portion of the PRF clot [19]. Therefore, we questioned to what extent adjusting the applied RCF could influence the cellular distribution within the solid PRF matrix, aiming to generate modified centrifugation protocols to enhance the regenerative capacity of PRF-based matrices. Thereby, fine tuning of the centrifugation process in terms of RCF reduction and slight increase of the centrifugation time resulted in an advanced PRF with enhanced leukocyte numbers, especially neutrophilic granulocytes. Moreover, the platelets and leukocytes within the advanced PRF were evenly distributed over the entire clot compared to PRF [19].

Based on these results, the question was raised to what extent a systematic reduction in RCF within fluid PRF matrices might have any influence on the increase of platelets and leukocytes as well as growth factors within the obtained blood concentrates.

In this context, the present study demonstrates a systematic analysis of the RCF influence on fluid PRF-based matrices. Based on the first introduced PRF, we halved the revolutions per minutes (rpm), which according to the radius of the respective centrifuge (110 mm) led to a reduction of the RCF by four times in a stepwise approach to examine a wide RCF range (i.e., 710–44 g) including the high, medium and low RCF, systematically. Simultaneously, the centrifugation time was kept constant to exclude its impact. To the best of our knowledge, the present study is the first to analyze the RCF impact on human blood concentrates to determine the leukocyte and platelet numbers as well as the growth factor release potential within PRF-based matrices. The aim of the study was to determine to what extent a controlled reduction of the RCF, by modifying the centrifugation setting rpm, could influence the platelet and leukocyte total number as well as the growth factor release within fluid PRF-based matrices.

## Materials and methods

### Systematic protocols for PRF-based matrices

To evaluate the high-, medium- and low-RCF spectrum, three different experimental centrifugation protocols for the systematic analysis series were established by decreasing the RCF within a wide range (710–44 g) with a constant centrifugation time. Based on PRF (708 g), a stepwise reduction of the rpm in half and, thus, a reduction of the RCF four times were performed for each protocol as follows:

I: 710 g; 2400 rpm; 8 min
II: 177 g; 1200 rpm; 8 min
III: 44 g; 600 rpm; 8 min

### PRF preparation

For this experiment, the blood of six healthy volunteers (three males and three females) was collected for each of the evaluated protocols. The tubes were immediately placed in the centrifuge and prepared according to the previously mentioned established protocols. A Duo centrifuge (Process for PRF, Nice, France) was used to perform the centrifugation procedure. This centrifuge has a fixed angle rotor with a radius of 110 mm. Written informed consent was obtained from the volunteers for their samples to be used in the research. All donors were free of any infectious disease and did not have any abnormal consumption of nicotine or alcohol. None of the subjects used any drugs for anticoagulation.

### Tubes for blood collection

For the purpose of these experiments, sterile plastic tubes (Process for PRF, Nice, France) with a volume of 10 ml were used to generate fluid blood concentrates according to the previously described protocols. This method was used because a fluid matrix was required for flow cytometry. The blood was drawn by means of a clinically approved butterfly blood collection method. The centrifugation for each protocol started after the last tube of this group was collected over a total time of 2–3 min maximum.

### Automated cell counting

The collected fluid matrices of each protocol were anti-coagulated using a BD vacutainer with 4 ml of ethylenediaminetetraacetic acid (EDTA). This anticoagulation was only performed for research purposes, as no cell counting measurements would be possible otherwise. The samples were further analyzed with ADVIA^®^ LabCell^®^ Automation Solution (Siemens, France) at a medical laboratory (Labazur laboratory, Nice, France) to detect the number of leukocytes and platelets per microliter. An automated cell count was performed by means of flow cytometry. This method enables a multiparameter analysis of the cell number suspended within a liquid. The cell suspension passes through a laser beam, where one cell per unit of time leads to laser scattering in different directions according to the cell sizes and properties. The scattered light is detected by a side and a forward sensor. The forward scatter is roughly proportional to the cell size, while side scatter is caused by the cell characteristics such as granularity and structural complexity [20]. These data are automatically further analyzed to detect the total number of leucocytes and platelets within the cell suspension, i.e., fluid PRF matrices.

### Growth factor quantification with ELISA

After centrifugation, the collected liquid PRF from each protocol was transferred into a cell culture plate. The plate was placed in 37 °C degree incubator until all the samples formed a clot. Afterwards, Dulbecco’s Modified Eagle Medium (Biochrom GmbH, Berlin, Germany) was added to all clots and further incubated in 37 °C degree to allow growth factor release. The supernatant (5 ml) was collected after 1 h and frozen. The collected supernatant was replaced by a fresh cell media and further incubated for 24 h. At the latter time point, the supernatants were collected. The collected samples at both time points were analyzed for human vascular epithelial growth factor (VEGF) and human transforming growth factor (TGF-β1). Protein quantification was performed by means of ELISA (DuoSet^®^ ELISA RND system) according to the manufacturer’s instructions. Optical density was assessed using a microplate reader at 450 and 570 nm. The data measured at 570 nm were subtracted from the data measured at 450 nm for an optical density correction. The measurements were performed in triplicates for each protocol and donor. Finally, the quantified data were statistically analyzed.

### Statistical analysis

Statistical analysis was performed using Prism Version 6 (GraphPad Software Inc., San Diego, La Jolla, USA). Data are expressed as the mean and standard deviation. The significance of the differences between the means was analyzed using one-way and two way analyses of variance (ANOVA) with Tukey multiple comparisons test (*α* = 0.05). Thereby, significant differences were marked as significant if *P* values were less than 0.05 (*P < 0.05) and highly significant if *P* values were less than 0.01 (**P < 0.01), 0.001 (***P < 0.001) or 0.0001 (****P < 0.0001).

## Results

### Total leukocyte number

**Fig. 1:**
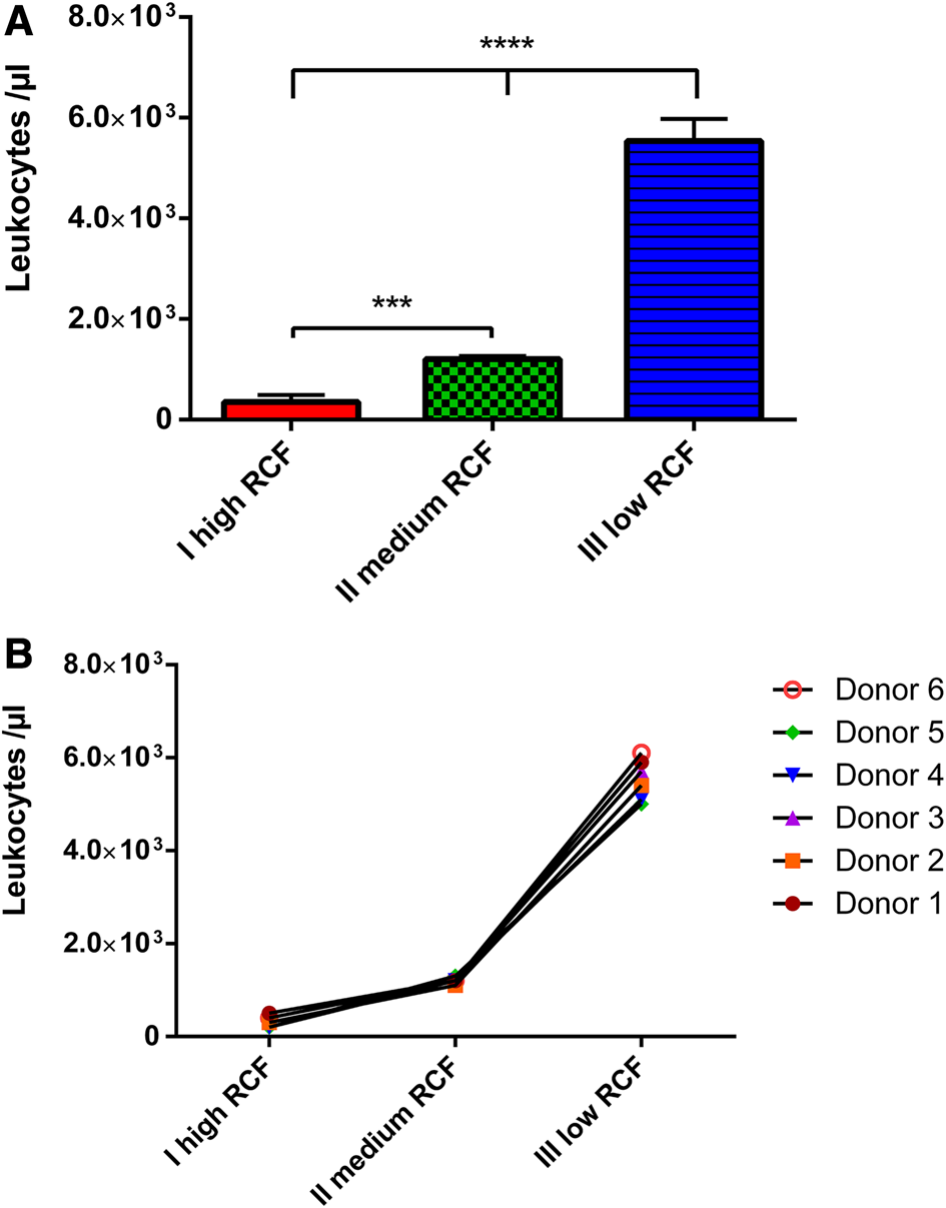
**a** Number of leukocytes within the different experimental PRF-based matrices. **b** Donor-related leukocyte number within the different experimental PRF-based matrices

The total leukocyte number was analyzed within the experimental PRF protocols. Generally, reducing the RCF led to a clearly detectable increase of the total leukocyte number within the PRF-based matrices. The first protocol-I (710 g), which was centrifuged with the highest RCF, showed the lowest number of leukocytes among the three evaluated experimental protocols. The second protocols I–II (177 g), using a four time slower RCF than protocol-I, showed a significantly higher number of leukocytes compared to protocol-I (*P* < 0.001). Finally, the third protocol-III (44 g) with four times less RCF than protocol-II and 16 times less RCF than protocol-I revealed the highest number of leukocytes, which was statistically highly significant compared to protocol-I (*P* < 0.0001) and protocol-II (*P* < 0.0001) (Fig. 1a).

The donor-related leukocyte total number showed similar results in each individual. All evaluated samples showed the same curve progression as a frequent observation of an increased leukocyte number with reduced RCF (Fig. 1b).

### Total platelet number

**Fig. 2:**
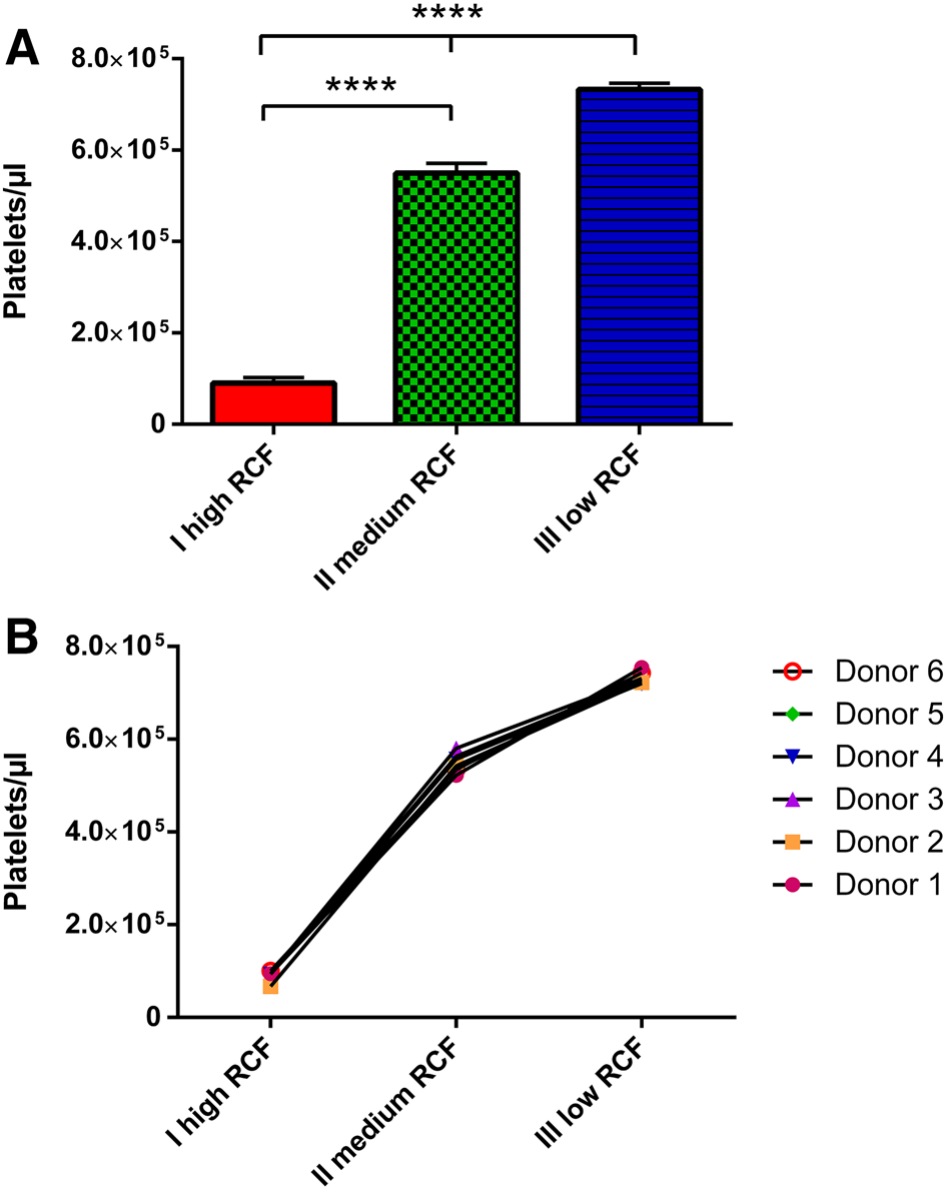
**a** Number of platelets within the different experimental PRF-based matrices. **b** Donor-related platelet number within the different experimental PRF-based matrices

The total platelet number as a result of automated cell counting showed a tendency towards increasing total platelet number with RCF reduction within the PRF-based matrices. The first experimental protocol-I (710 g) exhibited the lowest platelet number compared to all other examined groups. Looking at the second protocol-II (177 g), a significant increase in the platelet total number was detected in comparison to protocol-I (*P* < 0.0001). Moreover, a further RCF reduction resulted in the highest platelet total number in protocol-III (44 g). Statistical analysis showed significantly higher platelet numbers in protocol-III compared to protocol-II (*P* < 0.0001) and protocol-I (*P* < 0.0001) (Fig. 2a).

The donor-related values showed very similar reactions to the exposed RCF influence in the various PRF-based matrices. The curve shape was reproduced within the single donor samples, showing an increased number of platelets with reduced RCF (Fig. 2b).

### VEGF concentration

**Fig. 3:**
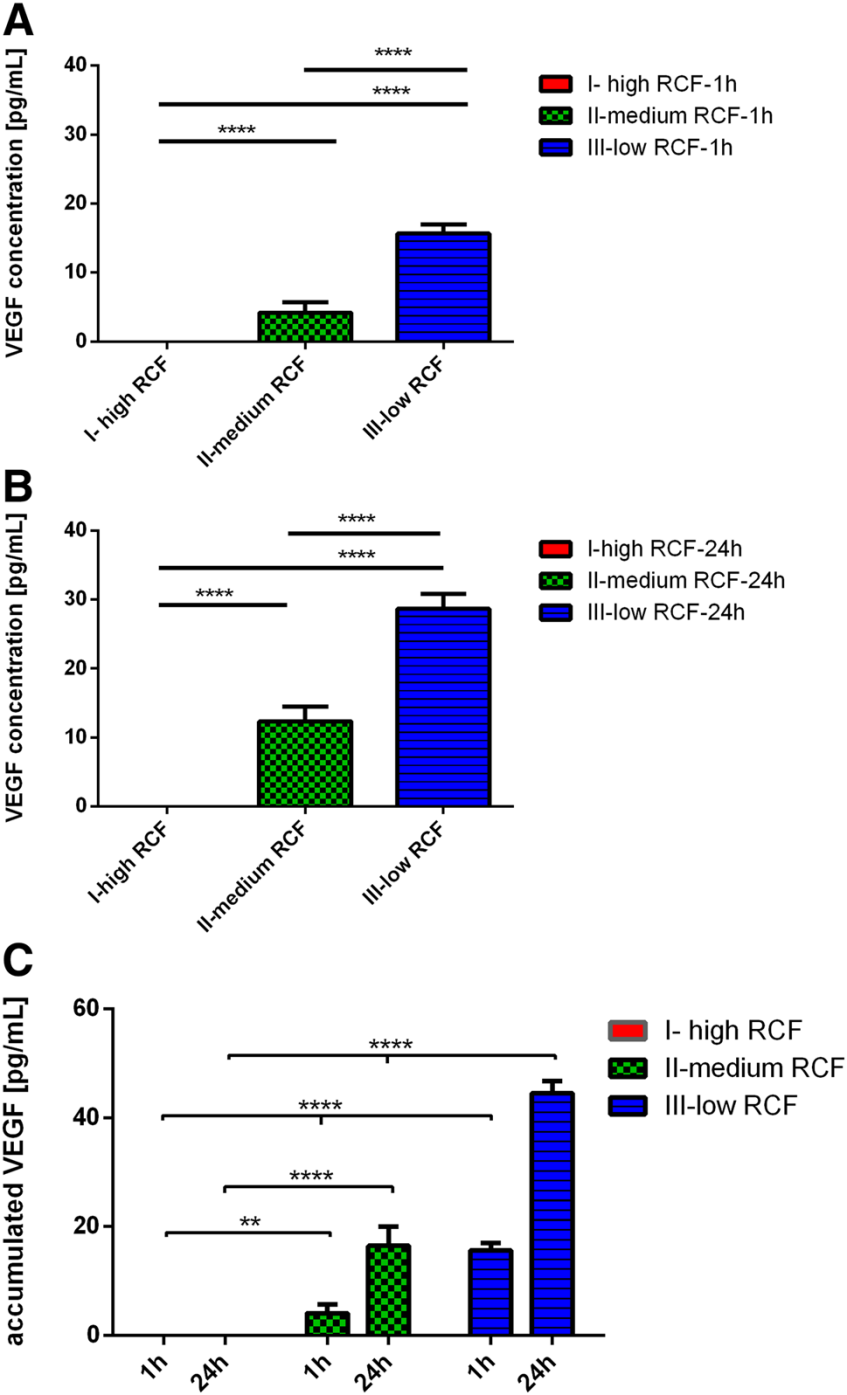
**a** VEGF concentration within the different experimental PRF-based matrices 1 h after clotting. **b** VEGF concentration within the different experimental PRF-based matrices 24 h after clotting. **c** Accumulated VEGF concentration within the different experimental PRF-based matrices over 24 h

The VEGF concentration was quantified 1 and 24 h after clotting. At both time points, a clear tendency was observed. The growth factor concentration increased by reducing the applied RCF. One hour after clotting, the VEGF concentration in protocol-I with the highest RCF showed the lowest concentration compared to the medium range RCF and low range RCF protocols. At the same time point, protocol-II, within the medium RCF range, showed increased VEGF concentration. These results were highly significant compared to protocol-I (*P* < 0.0001). Moreover, protocol-III with the lowest RCF application revealed the highest VEGF concentration. These data were highly significant compared to protocol-I (*P* < 0.0001) and protocol-II (*P* < 0.0001) (Fig. 3a). Similar results were detected 24 h after clotting. At this time point, protocol-I showed the lowest VEGF concentration. Along with RCF reduction, the VEGF concentration significantly increased in protocol-II. Statistical analysis showed a highly significant increase in protocol-II compared to protocol-I (*P* < 0.0001). Finally, protocol-III, which was prepared using the lowest RCF, showed the highest VEGF concentration which was highly significant compared to protocol-I (*P* < 0.0001) and protocol-II (*P* < 0.0001) (Fig. 3b).

The accumulated VEGF concentration over 24 h was calculated in the examined protocols. The released VEGF concentrations in all protocols increased from 1 to 24 h. At 24 h, the accumulated concentration in protocol-I was the lowest within the tested groups. Protocol-II showed a significantly higher accumulated VEGF concentration compared to protocol-I (*P* < 0.01). Additionally, the accumulated VEGF concentration in protocol-III was the highest, which was highly significant compared to protocol-I (*P* < 0.0001) and protocol-II (*P* < 0.0001) (Fig. 3c).

### TGF-β1 concentration

**Fig. 4:**
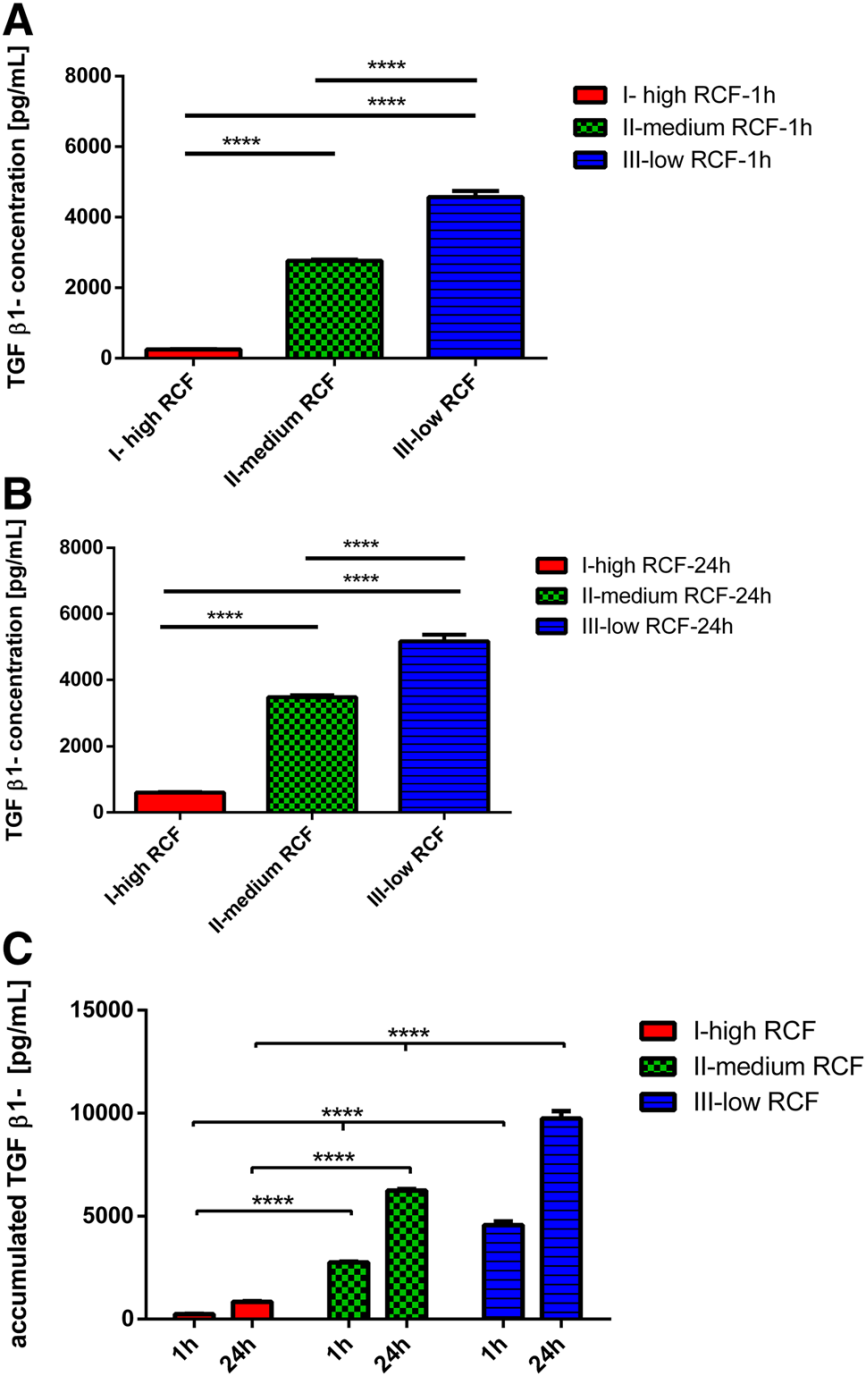
**a** TGF β-1 concentration within the different experimental PRF-based matrices 1 h after clotting. **b** TGF β-1 concentration within the different experimental PRF-based matrices 24 h after clotting. **c** Accumulated TGF β-1 concentration within the different experimental PRF-based matrices over 24 h

The growth factor concentration for human TGF-β1 was measured 1 and 24 h after clotting. A general trend was observed at both time points. Reducing the applied RCF enhanced the growth factor concentration. Looking at the results 1 h after clotting, protocol-I showed the lowest TGF-β1 concentration among all tested protocols. Thereby, protocol-II, which was prepared within the medium RCF range, revealed a significantly higher TGF-β1 concentration when compared to protocol-I (*P* < 0.0001), which was prepared within the high RCF range. Additionally, protocol-III, representing the low RCF range, showed the highest TGF-β1 concentration among the analyzed protocols. This concentration was highly significant compared to protocol-I (*P* < 0.0001) and protocol-II (*P* < 0.0001) (Fig. 4a).

Twenty-four hours after clotting, the TGF-β1 was detectable in all protocols. However, protocol-I showed the lowest concentration compared to protocols-II and -III. The first step RCF reduction toward protocol-II within the medium RCF range showed a highly significant increase of TGF-β1 concentration compared to the high RCF range in protocol-I (*P* < 0.0001). Further RCF reduction toward the low RCF range in protocol-III reached the highest TGF-β1 concentration among all tested protocols. Thereby, this increase was highly significant compared to protocol-I (*P* < 0.0001) and protocol-II (*P* < 0.0001) (Fig. 4b).

The accumulated TGF-β1 concentration increased from 1 to 24 h in all protocols. However, protocol-I showed the lowest accumulated TGF-β1 concentration among all tested protocols. Whereas in protocol-II, which was prepared within the medium RCF range, a significantly higher accumulated TGF-β1 level compared to protocol-I was observed. Finally, protocol-II, which represents the low RCF range, showed the highest accumulated TGF-β1 concentration. This value was statistically highly significant compared to protocols-I and -II (Fig. 4c).

## Discussion

The present study analyzed the platelets and leukocyte total number, as well as the growth factor release within fluid PRF matrices with different preparations of settings. To the best of our knowledge, this study is the first to investigate the influence of RCF on the cell number and growth factor release in fluid PRF-based human blood matrices. Using specific experimental centrifugation protocols, which were systematic modifications of the first described PRF [13], we generated PRF-based fluid matrices with different proportions of platelets and leukocytes. Moreover, a relation between RCF reduction and growth factor release was evidenced. The present data provide new insight into the potential of the centrifugation process in generating different total cell numbers and growth factor levels of release within the same blood volume only in relation to the amount of exposure to specific RCF.

Three experimental protocols were established with a constant centrifugation time to focus on the impact of RCF across various ranges, including high, medium and low. Adapting the RCF spectrum (710–44 g) based on the first described PRF protocol [13], a systematic reduction of the RCF was performed by a four time RCF reduction for each protocol (I–III) as a consequence of stepwise halving the rpm (revolutions per minute), because the centrifuge radius used in this study was 110 mm.

Automated cell count by means of flow cytometry was performed to determine the total leukocyte and platelet number. The results showed that decreasing RCF up to 16 times less than the first described PRF led to a significant increase in leukocyte and platelets numbers within the generated PRF-matrices. The first protocol-I (710 g) showed the lowest detectable number of leukocytes and platelets among all samples tested. Interestingly, reduction of RCF in protocol-II (177 g) resulted in a significantly higher number of leukocytes and platelets compared to protocol-I (710 g). Moreover, the second step RCF reduction toward protocol-III (44 g) revealed a further significant increase of the leukocyte and platelet total number—with the highest value in comparison to all other groups. These data emphasize that modifications of RCF contribute to a clearly significant alteration of the leukocyte and platelet number toward the lower RCF ranges (177–44 g).

Centrifugation is a widely used technique to separate a biological mixture within a liquid phase. The principles of this technique are based on the use of the centrifugal force, which is much higher than the gravity. During centrifugation, different forces interact and influence the particle movement within the liquid including centrifugal force, gravitational force and the drag force of the particles, i.e., cells. This process results in particle migration depending on their size, density and mass [21]. The present results suggest that the mass, size and density range of leukocytes and platelets require a low RCF, which is enough to separate them from the rest blood components while not causing aggregation to the bottom of the tube. However, further studies are needed to demonstrate to what extent higher or lower RCF ranges than the presently investigated spectrum may have on any further benefits regarding cell and growth factor accumulation within the fluid PRF matrices. Thus, the RCF reduction can be used as a “tool” to generate fluid PRF-based matrices enriched with leukocytes and platelets. This phenomenon is of high scientific and clinical relevance, as leukocytes are one of the main drivers of bone and soft tissue regeneration by contributing to the release of the angiogenic and lymphogenic factors responsible for cellular cross-talk in the tissue regeneration process [18, 22]. Leukocytes are also known to be involved in the communication between precursor cells and mesenchymal cells with regard to bone formation [23–26]. Accordingly, without leukocytes, sophisticated cell–cell communication for tissue regeneration is not possible. In addition, platelets are known to harbor potent growth factors (PDGFs) and platelet-derived growth factors for tissue regeneration such as vascular endothelial growth factor (VEGF) and transforming growth factor-beta (TGF-ß) [17, 27–29], which can be released only after platelet aggregation [30, 31]. In addition, platelets are not the only players in tissue regeneration—they require leukocytes for better performance in their capacity towards tissue regeneration [32–34].

Furthermore, the present study demonstrated that systematic reduction of the applied RCF results in a clearly increased tendency in growth factor concentration. Thus, the determined growth factors VEGF and TGF-β1 were the lowest in protocol-I, which was prepared within the high RCF range. However, RCF reduction toward the medium RCF range led to a significant increase of VEGF and TGF-β1 concentrations in protocol-II. Furthermore, the RCF reduction showed the highest VEGF and TGF-β1 in protocol-III within the low RCF spectrum. This observation was evidenced at 1, 24 h as well as for the accumulated growth factor over 24 h. These findings are in correlation with the results shown by increasing the number of platelets and leukocytes. In this context, the increased growth factor concentration within the medium and low RCF ranges is probably related to the increased number of platelets and leukocytes as these cells are important sources of growth factors. Moreover, it might be that applying a high RCF not only results in a lower number of platelets and leukocytes but also affects these cells’ ability to release growth factors. Consequently, enhancing the growth factor concentrations within the fluid PRF-based matrices reflects improving the regenerative capacity of the fluid PRF-based matrices as an autologous growth factor reservoir. VEGF is one of the most important signaling molecules for neoangiogenesis, which is highly required during wound healing [35]. Moreover, TGF-β1 contributes to tissue regeneration rendering the recruitment of keratinocytes, especially in the early wound healing stage [36–38].

To date, there are no data showing how many leukocytes or platelets within the PRF-based matrices are sufficient to have the best possible physiological condition according to which an optimal condition for wound healing or a basis for a successful soft tissue and bone regeneration can be generated. The results of the present study highlight, however, that RCF reduction might be a clinical applicable tool, in order to tailor the amount of leukocytes within the PRF-derived blood concentrates according to the required indication.

Based on the present data, according to which reduction of RCF results in PRF-matrices enriched with leukocytes and platelets and enhanced growth factor release, we postulated the so-called LSCC, which can be used to generate fluid PRF-based matrices enriched with cells and plasma proteins from peripheral blood as an autologous source for a smart tissue regeneration in complex tissue engineering.

In a clinical setting, there is a need to increase the regenerative potential of bone substitute materials or bio-membranes. This could be achieved by adding autologous tissue engineering fluid systems. Accordingly, the presented plastic tubes and the RCF reduction allowed for the generation of a liquid injectable PRF-based matrices (i-PRF) without the use of anticoagulants. This i-PRF, prepared according to the LSCC, is highly enriched with platelets, leukocytes and growth factors, which could provide a significant benefit for the regeneration process.

Recently, our group has shown that the addition of only monocytes isolated from peripheral blood contributes to increasing the in vivo vascularization of synthetic bone substitute materials [39]. Consequently, looking at PRF as a complex “physiological” system, which can be generated by a one step centrifugation, it is assumed that in this system monocytes, and all other substances and cells can be enriched. This system might, therefore, be able to contribute to an improved wound healing condition along with enhanced vascularization, while remaining within their cell-specific niche. These data underline that fluid PRF-based matrices, gained by reduced RCF, can be used to functionalize biomaterials by means of an autologous source, i.e., concentrates of peripheral blood to promote tissue regeneration.

Recent ex vivo work by our group demonstrated that alerting the RCF within solid PRF-based matrices advances the cellular distribution and enhances the neutrophilic granulocytes number as a leukocytes subgroup within the advanced PRF clot [19]. However, further studies are necessary to show the role of RCF reduction on cell number and growth factor release in solid PRF-based matrices.

The blood composition is specific and individual according to the particular donor. Still, the donor-related values within the different PRF-based matrices were approximately similar in all groups regarding the platelet distribution as well as the leukocytes. These findings indicate that first of all, PRF-based matrices are reproducible systems and individually applicable regardless of the donor characteristics. Second, the results also establish the reproducibility of the LSCC in various blood samples when comparing the six different blood donors.

In this study, the data demonstrate that it is possible to apply the LSCC for the enrichment of blood concentrates with platelets, leukocytes and growth factors. The combination of leukocytes and platelets plays an important role in tissue regeneration [40, 41]. Thus, the ability to control the cell content within blood-derived fluid PRF-based matrices by only changing the centrifugation settings, such as rpm, i.e., changing the exposure to a specific RCF, might serve as a valuable step to have personalized medicine for widespread clinically applicable cell-based tissue engineering. Thereby, using RCF as a tool to control the number of the included cells could be used to adjust the preparation protocol to the specific needs of individual patients according to the clinical indications.

Based on the present outcome, the LSCC helps to show that the number of several blood components responsible for tissue regeneration, i.e., platelets, leukocytes and growth factors, can be selectively enriched by application of a clinically applicable system through a single parameter within the centrifugation process. Considering the complexity of cell isolation and cultivation in laboratories under sterile conditions, the PRF system embodies a relatively simple tool to influence the number of these cells. In this context, further systematic in vivo and clinical studies that evaluate the benefit of this system, i.e., enhancing the autologous regeneration capacity, are needed to assess the correlation between cell enrichment and the improved potential of tissue regeneration and wound healing.

## Conclusions

In the present study, the growth factor release and the leukocyte and platelet total numbers were analyzed in relation to the systematic variation of the relative centrifugation force (RCF) exposure for the first time. The present data demonstrated that reducing the RCF from a high range toward a low spectrum within autologous PRF-based matrices leads to a significant increase of the leukocyte and platelet number, as well as growth factor concentration (VEGF and TGF-β1). Based on these results, we postulate the low speed centrifugation concept (LSCC) enhances the regeneration potential of fluid PRF-based matrices. Consequently, the reduction of RCF by application of LSCC opens up new avenues for advanced PRF-matrices, in which the cell–cell communication between platelets and leukocytes and that of these cells within the recipient tissue might result in improved wound healing and enhanced tissue regeneration. Thus, further preclinical and clinical studies are necessary to evaluate this concept to optimize clinical benefits.

## Abbreviations

PRF: Platelet-rich-fibrin
LSCC: Low speed centrifugation concept
